# Capsaicin suppresses interleukin-31-induced itching partially involved in inhibiting the expression of dorsal root ganglion interleukin-31 receptor A in male mice

**DOI:** 10.1016/j.ynpai.2022.100088

**Published:** 2022-03-29

**Authors:** Iwao Arai, Minoru Tsuji, Hiroshi Takeda, Nobutake Akiyama, Saburo Saito

**Affiliations:** a Department of Pharmacology, International University of Health and Welfare, 2600-1, Kitakanemaru, Ohtawara, Tochigi 324-8510, Japan; b Core Research Facilities for Basic Science, Research Center for Medical Science, The Jikei University School of Medicine, 3-25-8 Nishi-Shinbashi, Minato-ku, Tokyo 105-8461, Japan; c Division of Environmental Allergy, The Jikei University School of Medicine, 3-25-8 Nishi-Shinbashi, Minato-ku, Tokyo 105-8461, Japan

**Keywords:** **IL-31**, Interleukin-31, **IL-31RA**, Interleukin-31 receptor A, **TRPV1**, transient receptor potential vanilloid member 1, **AD**, Atopic dermatitis, **DRG**, dorsal root ganglia, Capsaicin, Interleukin-31 (IL-31), IL-31 receptor A (IL-31RA), Itch, Transient receptor potential vanilloid member 1 (TRPV1)

## Abstract

To elucidate the mechanisms underlying the antipruritic effect of capsaicin, we investigated how topical application of capsaicin (0.01, 0.1 and 1.0% w/v) affects spontaneous scratching in NC/Nga mice, inerleukin-31 (IL-31) induced in BALB/c mice, and IL-31 receptor A (IL-31RA) and transient receptor potential vanilloid member 1 (TRPV1) mRNA expression in dorsal root ganglia (DRG). Capsaicin concentration-dependently suppressed long-lasting scratching (over 1.0 s, itch-associated scratching) and short-lasting scratching (0.3–1.0 s, locomotor activity) immediately after the application. Total long-lasting scratching and short-lasting scratching counts for 24 h and IL-31RA mRNA expression in the DRG significantly decreased with increasing concentration of capsaicin. Furthermore, 1.0% capsaicin suppressed long-lasting scratching and short-lasting scratching for more than 72 h. At this point, DRG IL-31RAmRNA was significantly decreased, but there was no change in cutaneous IL-31RA and TRPV1 mRNA. Thus capsaicin suppresses long-lasting scratching by inhibiting IL-31RA mRNA expression in the DRG. Next, we examined the effect of capsaicin on IL-31-induced long-lasting scratching in BALB/c mice. Repeated administration of IL-31 (50 μg/kg, subcutaneous) every 12 h for 3 days apparently increased long-lasting scratching counts and IL-31RA mRNA in the DRG. These increases were significantly suppressed by pretreatment with 1.0% capsaicin. TRPV1 mRNA in the DRG was also decreased within 1–24 h after capsaicin application. These results suggest that the strong and prolonged antipruritic action for IL-31-induced itching of capsaicin was caused by desensitization of C-fibers, and, in addition, the long-lasting inhibition of IL-31RA mRNA expression in the DRG.

## Highlights

• Topically applied capsaicin suppressed scratching behavior in NC/Nga mice, an animal model of atopic dermatitis, sustained for more than 72 h after application.
• Topically applied capsaicin suppressed IL-31-induced scratching behavior in BALB/c mice, sustained for more than 72 h after application.
• Topically applied capsaicin suppressed IL-31receptor A mRNA expression in the DRG, sustained for more than 72 h after application.
• This is the first report that an inhibitor of IL-31receptorA expression suggests a possible mechanism for atopic dermatitis treatment.

## 1 Introduction

In a previous study, spontaneous scratching was measured in NC/Nga mice (Takano et al. 2003), an animal model of atopic dermatitis (Matsuda et al. 1997). We had previously demonstrated that IL-31 gene transcripts were significantly increased in skin-lesioned NC/Nga mice, and this change coincided with an increases in itch-associated scratching (long-lasting scratching) counts (Takaoka et al. 2005). Moreover, repeated administration of IL-31 significantly increased long-lasting scratching and IL-31RA expression in the DRG, and long-lasting scratching was closely correlated with IL-31RA mRNA expression in the DRG (Arai et al., 2014, Arai et al., 2015). NC/Nga mice developed severe spontaneous long-lasting scratching and dermatitis. Therefore, IL-31 among the most important factor that induced itching and promote itching in skin-lesioned NC/Nga mice. Similarly, reported administration of IL-31 in BALB/c mice every 12 h for 3 days gradually increased long-lasting scratching and IL-31RA mRNA in the DRG expression which reached a plateau after the 3 days (Arai et al. 2016). There were close correlations between long-lasting scratching and IL-31RA mRNA expression in the DRG. We postulated that IL-31 stimulates IL-31RA mRNA expression and increases IL-31RA protein production in the DRG neuronal cell bodies, and cutaneous IL-31-induced itching is enhanced by DRG IL-31RA expression in mice (Arai et al. 2016). Therefore, we searched for inhibitors that suppress itching and evaluated various agents by using spontaneous long-lasting scratching counts as the indicator in skin-lesioned NC/Nga mice in order to develop the therapeutic drugs for atopic dermatitis (AD). Eventually, we discovered that the topical application of capsaicin significantly suppressed long-lasting scratching counts in skin-lesioned NC/Nga mice and determined that capsaicin is among the strongest inhibitors of long-lasting scratching (Takano et al. 2004).

Capsaicin is known to act on the transient receptor potential cation channel vanilloid subfamily V member 1 (TRPV1). TRPV1 is involved in the modulation of nociceptive inputs to the spinal cord and brain stem centers, as well as the integration of diverse painful stimuli (Frias and Merighi, 2016). The sensation of itching can be reduced by the painful sensations caused by scratching (Chung et al. 2011). The inhibition of itching by painful stimuli has been experimentally demonstrated using various painful stimuli. We previously demonstrated that cutaneous prostaglandins (PGs) levels were significantly elevated when mouse skin was scratched with a stainless steel wire brush (mechanical scratching) (Futaki et al. 2007), and these PGs suppressed long-lasting scratching in skin-lesioned NC/Nga mice (Arai et al. 2004). As PGs are associated with inflammation, their administration was found to enhanced pain (Arai, et al., 2016). Surprisingly, although pain-induced suppression of itching is a temporary action, this effect of capsaicin continues for more than 72 h. Thus this effect may not only be due to pain but also another action of capsaicin. Therefore, in this study, we elucidated the mechanism of action of capsaicin further by investigating whether there was a relation between IL-31 and IL-31RA and TRPV1 mRNA expression in the DRG.

## 2 Methods

### 2.1 Animals

Male 13-week-old skin-lesioned NC/Nga mice and 8-week-old BALB/c mice were purchased from SLC Japan (Shizuoka, Japan). The animals were housed under conditions of controlled temperature (23 ± 1 °C), humidity (50 ± 2%) and lighting (lights on from 7:00 am to 7:00 pm). All animals were given free access to food and tap water. All procedures for animal experiments were approved by the Committee for Animal Experimentation at the International University of Health and Welfare and were in accordance with the Guidelines for Proper Conduct of Animal Experiments (Science Council of Japan, 2006) and the guidelines of the International Association for the Study of Pain (Zimmermann 1983).

### 2.2 Reagents

Mouse IL-31 cDNA spanning amino acids 24–163 of IL-31 was cloned in-frame with pET30A (Novagen, Darmstadt, Germany), and the construct was transformed in BL-21 cells (Novagen). After induction with isopropyl-β-D-thiogalactopyranoside, IL-31 protein was purified under denaturing conditions using nickel-chelating sepharose (Qiagen, Benelux B.V. Netherlands), and dialyzed in phosphate buffer solution (Akiyama et al. 2009b). Capsaicin (Tokyo Kasei, Osaka, Japan) was dissolved in ethanol (Kokusan kagaku, Tokyo, Japan), and 200 μl of this solution was applied to the rostral part of the back of the mice.

### 2.3 Measurement of scratching counts

Scratching was measured as described previously (Arai et al. 2015). Two kinds of scratching behavior were observed: long-lasting scratching (over 1.0 s) and short-lasting scratching (0.3 – 1.0 s). In a previous study, long-lasting scratching was frequently seen in spontaneous skin-lesioned NC/Nga mice, but not in other strains of mice. In contrast, short-lasting scratching was frequently seen in both skin-lesioned NC/Nga mice and other strains of mice. These results suggest that short-lasting scratching is a form of social and/or hygienic behavior, while long-lasting scratching is the true itching response in these mice. Therefore, we investigated long-lasting scratching as an indicator of itching (Takano et al. 2004). The number of scratches was detected automatically and evaluated objectively using MicroAct (Neuroscience, Tokyo, Japan). The parameters for detecting waves were threshold, 0.1 V; event gap, 0.2 s; minimum duration, 1.5 s; maximum frequency, 20 Hz; and minimum frequency, 2 Hz.

### 2.4 Hot-plate test

The hot-plate test (Eddy and Leimbach, 1953) was used to measure the latency to paw withdrawal as described previously (Tsuji et al., 2019). This experiment was not increase pain threshold. The mice were placed on a hot-plate and the time (latency) until either a paw-lick response or an attempt to escape by jumping was recorded. The mice were tested before the application of 1.0% capsaicin which was applied to the limbs of the mouse for reducing the pain threshold at low temperature (maintained at 35.0 ± 0.3 °C), and 1, 6, 24 and 72 h after application. To prevent tissue damage, mice that showed no response within 60 s were removed from the hot-plate and assigned a score of 60 s.

### 2.5 Quantitative real-time polymerase chain reaction

The expression of IL-31, IL-31RA, TRPV1 and β- actin was determined by real-time polymerase chain reaction (RT-PCR) in the DRG (C_4-7_, T_1-4_) neuron cell body from the shoulder and back of NC/Nga and BALB/c mice at each point. Total RNA was extracted from the dorsal skin of each mouse by Trizol (Invitrogen, Carlsbad, CA,USA) and digested using amplification-grade DNase I (Invitrogen), according to the manufacturer’s instruction. cDNA was synthesized by the SuperScript III First-Strand Synthesis System (Invitrogen). Quantitative RT-PCR was performed with SYBR Green Master Mix, using an Applied Biosystems 7700 Sequence Detection System (Applied Biosystems, Foster City, CA, USA). The PCR primers for IL-31 were designed using Primer 3 software, and primers for TRPV1 and β- actin were purchased from TAKARA BIO (Otsu, Shiga, Japan). Primer sequences were as follows: IL-31 (’5-ATA CAG CTG CCG TGT TTC AG-3′ and 5′-AGC CAT CTT ATC ACC CAA GAA-3′), IL-31RA (‘5-CCA GAA GCT GCC ATG TCG AA-3′ and 5′-TCT CCA ACT CGG TGT CCC AAC-3′), TRPV1 (‘5-CAA CAA GAA GGG GCT TAC ACC-3′ and 5′-TCT GGA GAA TGT AGG CCA AGA C-3′) and β - actin (5′-TGA CAG GAT GCA GAA GGA GA-3′ and 5′-GCT GGA AGG TGG ACA GTG AG-3′). Relative expression levels were calculated using the relative standard curve method as outlined in the manufacturer’s technical bulletin. A standard curve was generated using the fluorescence data obtained from four-fold serial dilutions of the total RNA of the sample with the highest expression. The curve was then used to calculate the relative amounts of target mRNA in test samples. Quantities of all targets in the test samples were normalized to the corresponding β- actin RNA transcript in skin samples.

### 2.5 Data analysis

All data were statistically analyzed using GraphPad InStat software (GraphPad InStat Inc., La Jolla, CA, USA). Experimental values are expressed as means and standard errors (SE) Student’s unpaired *t*-test, Dunnett test or Tukey’s multiple comparison test were used to compare data from the scratching counts. Values of *P* < 0.05 were considered statistically significant.

## 3 Results

### 3.1 Effect of topical application of capsaicin on spontaneous scratching behavior of skin-lesioned NC/Nga mice

**Fig. 1:**
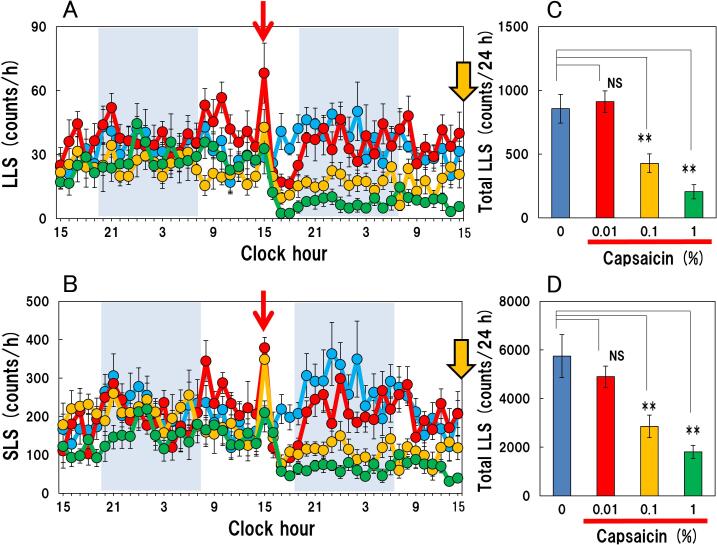
Effect of topically applied capsaicin on spontaneous scratching behavior of skin-lesioned NC/Nga mice (A) Typical intra-day pattern outlining the inhibitory effect of topically applied capsaicin (0.01, 0.1 and 1.0% w/v) on long-lasting scratching of NC/Nga mice. The blue line indicates the vehicle- treated group; red line indicates the 0.01% capsaicin-treated group; yellow line indicates the 0.1% capsaicin treated-group; green line indicates the 1.0% capsaicin treated-group. The red arrow indicates the application of vehicle or capsaicin. The yellow arrow indicates the sampling point of PCR for Fig. 2. Data represent scratching counts at each hour. The lateral axis indicates the clock hour, and the shaded area represents the dark phase (7:00 pm to 7:00 am). (B) Typical intra-day inhibition pattern of topically applied capsaicin on short-lasting scratching (social behavior) of NC/Nga mice. (C) The total long-lasting scratching counts for 24 h of capsaicin. The blue column indicates vehicle treated group; the red column indicates 0.01% capsaicin-treated group; the yellow column indicates 0.1% capsaicin-treated group; the green column indicates 1% capsaicin-treated group. (D) Total short-lasting scratching counts after 24 h applying capsaicin for 24 h. Each value represents the mean ± SE from six mice. NS, not significant, **P < 0.01 when compared with the vehicle-treaed (0) group (Dunnett test). (For interpretation of the references to colour in this figure legend, the reader is referred to the web version of this article.)

Topically applied capsaicin (0.01, 0.1 and 1.0% w/v) suppressed long-lasting scratching (Fig. 1A) and short-lasting scratching (Fig. 1B) counts in NC/Nga mice immediately after application in a concentration dependent. The suppressive action of 0.01% of capsaicin subsided 6 h after its application, while the suppressive action of 0.1 and 1.0% of capsaicin was sustained for more than 24 h. The total long-lasting scratching counts in the mice after administering 0 (vehicle), 0.01, 0.1 and 1.0% of capsaicin topically were 855.4 ± 112.4, 911.3 ± 84.7, 427.4 ± 73.3 and 204.7 ± 55.4 counts/24 h, respectively (Fig. 1C). Total long-lasting scratching was significantly suppressed by treatment with 0.1 and 1.0% of capsaicin but not 0.01%, compared with vehicle-treated mice. The total short-lasting scratching counts for 24 h after administering 0 (vehicle), 0.01, 0.1 and 1.0% of capsaicin topically were 5746 ± 885, 4899 ± 437, 2857 ± 461 and 1806 ± 265 counts/24 h, respectively (Fig. 1D). Total short-lasting scratching was also significantly suppressed by treatment with 0.1 and 1.0% of capsaicin but not 0.01%, compared with vehicle-treated mice.

### 3.2 Dose dependent inhibitory effect of topically applied capsaicin on DRG IL-31RA mRNA expression in skin-lesioned NC/Nga mice

**Fig. 2:**
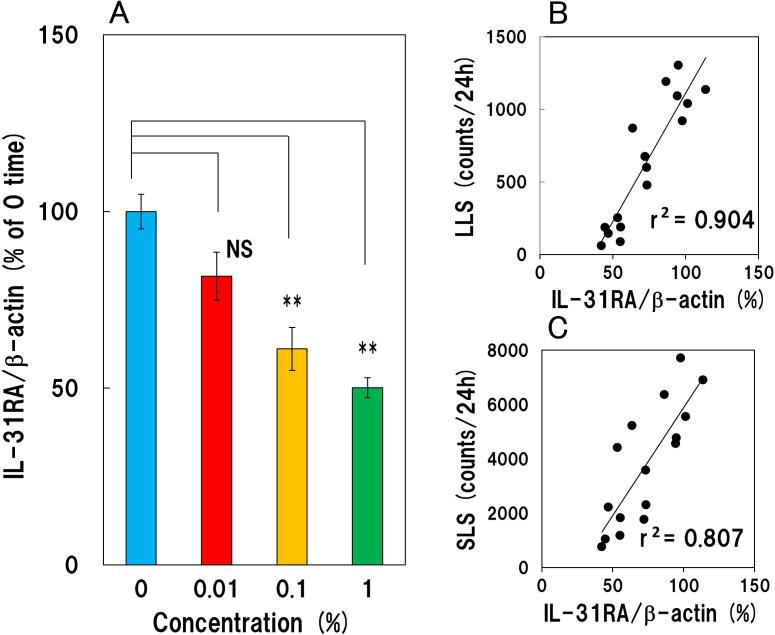
Dose dependent inhibitory effect of topically applied capsaicin on IL-31RA mRNA expression in NC/Nga mice (A) IL-31RA mRNA expression in the dorsal root ganglia (DRG). The blue column indicates the vehicle- treated group, the red column indicates the 0.01% capsaicin-treated group, the yellow column indicates the 0.1% capsaicin-treated group, the green column indicates the 1.0% capsaicin-treated group. (B) correlation 1: correlation between the IL-31RA mRNA expression in the DRG and long-lasting scratching in skin-lesioned NC/Nga mice. (C) correlation 2: correlation between the IL-31RA mRNA expression in the DRG and short-lasting scratching in skin-lesioned NC/Nga mice. Each value represents the mean ± SE from four mice. NS, not significant, **P < 0.01 when compared with the vehicle treated (0) group (Dunnett test). (For interpretation of the references to colour in this figure legend, the reader is referred to the web version of this article.)

After 24 h the expression of IL-31RA mRNA in the DRG of 0.01, 0.1 and 1.0% capsaicin was 81.7 ± 6.8, 61.1 ± 6.1 and 50.1 ± 2.8% (% of control). Topically applied capsaicin (0.1 and 1.0%) significantly suppressed IL-31RA mRNA expression in the DRG, compared with vehicle-treated mice but not 0.01% (Fig. 2A). The decreased IL-31RA mRNA expression in the DRG was significantly correlated with decreased long-lasting scratching (Fig. 2B, r = 0.904) and short-lasting scratching counts (Fig. 2C, r = 0.807).

### 3.3 Time course of the changes in the effects of capsaicin on scratching behavior, cutaneous IL-31RA mRNA and IL-31RA, TRPV1 mRNA expression in the DRG in skin-lesioned NC/Nga mice

**Fig. 3:**
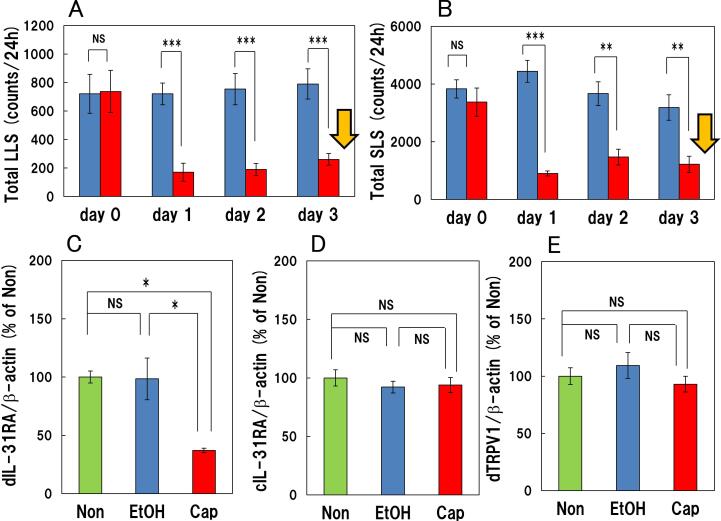
Effect of administering high concentration of capsaicin 72 h after topically application on spontaneous scratching behavior and on cutaneous and IL-31RA and IL-31RA and TRPV1 mRNA expression in the DRG in skin-lesioned NC/Nga mice (A) Total long-lasting scratching and (B) short-lasting scratching counts of vehicle and capsaicin treated group. The blue column indicates the vehicle-treated group. The red column indicates the 1.0% capsaicin-treated group. Day 0 indicates the time before vehicle or capsaicin treatment, while day 1, 2, 3 indicates 0–24 h, 24–48 h, 48–72 h, respectively, after vehicle or capsaicin treatment in each group. The yellow arrow indicates the sampling point of PCR for Fig. 3C - E. Each value represents the mean ± SE from six mice. NS, **P < 0.01 and ***P < 0.001 when compared with the vehicle treated group (Student’s *t* test). (C) IL-31RA mRNA in the DRG. (D) Expression of cutaneous IL-31RA mRNA. (E) TRPV1 mRNA in the DRG. The green column indicates non-treated group, the blue column indicates vehicle-treated group, the red column indicates 1.0% capsaicin-treated group. *P < 0.01 when compared with the vehicle (ethanol: EtOH) treated group (Tukey’s test). (For interpretation of the references to colour in this figure legend, the reader is referred to the web version of this article.)

There were no changes in the long-lasting scratching and short-lasting scratching counts during the experimental period from day 0 (-24–0 h) after pretreatment to day 3 (0–72 h) after treatment in the group treated with vehicle (Fig. 3A, B, blue column). In contrast, topically applied capsaicin (1.0%) significantly suppressed long-lasting scratching and short-lasting scratching counts from immedia**t**ely after treatment to day 1 (0–24 h), day 2 (24 – 48 h) and day 3 (48–72 h) after treatment compared with the pretreatment counts of the day 0 (Fig. 3A, B; red column) in skin-lesioned NC/Nga mice. The total long-lasting scratching counts on days 0 (pretreatment), 1, 2 and 3 measured at 24 h-intervals after applying 1.0% capsaicin were 738.0 ± 147.6, 170.6 ± 62.8, 189.5 ± 42.6 and 261.2 ± 41.8 counts/24 h, respectively (Fig. 3A). The total short-lasting scratching counts for 24 h of day 0 (pretreatment), day 1, day 2 and day 3 after applied of capsaicin were 3375 ± 487, 907 ± 82, 1472 ± 270 and 1220 ± 283 counts/24 h, respectively (Fig. 3B).

In the vehicle (ethanol, EtOH) treated group, there were no changes in the cutaneous and DRG IL-31RA and DRG TRPV1 mRNA expression after 0–72 h following treatment compared with the non-treatment group (Non, Fig. 3 C - E, green and blue columns). IL-31RA mRNA expression in the DRG was significantly decreased at 0–72 h after treatment with 1.0% capsaicin (Fig. 3C, red column).

In contrast, there were no changes in the cutaneous IL-31RA and DRG TRPV1 mRNA expression at 72 h after treatment with 1.0% capsaicin (Fig. 3D - E, red column).

### 3.4 Effect of topically applied capsaicin on IL-31-induced scratching behavior in BALB/c mice

**Fig. 4:**
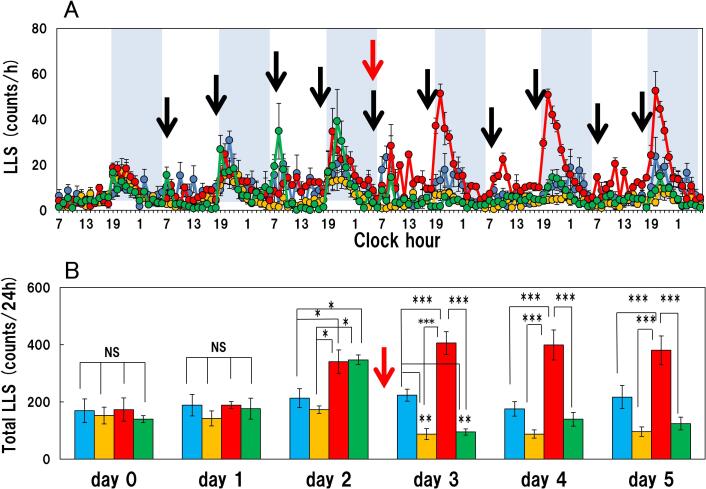
Effect of topically applied 1.0% capsaicin on IL-31-induced itch-associated scratching behavior (long-lasting scratching) in BALB/c mice. (A) Long-lasting scratching induced by IL-31 was measured for 96 h in BALB/c mice. The black arrow indicates the site of injection of the vehicle or IL-31 (50 μg/kg, subcutaneous) every 12 h (7:00 am and 7:00 pm) for 96 h. The lateral axis indicates the clock hour, and the shaded area represents the dark phase (7:00 pm to 7:00 am). The red arrow indicates the site of topical application of the vehicle or capsaicin. The blue line indicates saline + vehicle-treated group, the yellow line indicates saline + capsaicin treated group; the red line indicates IL-31 + vehicle-treated group; the green line indicates the IL-31 + capsaicin-treated group. (B) Total long-lasting scratching counts measured 24 h before administering IL-31 (day 0) and on day 1 (0–24 h), day 2 (25–48 h), day 3 (48–72 h), day 4 (72–96 h) and day 5 (96–120 h) after administering IL-31. The blue column indicates saline + vehicle-treated group, the yellow column indicates saline + capsaicin treated group; the red column indicates IL-31 + vehicle-treated group; the green column indicates IL-31 + capsaicin-treated group. Each value represents the mean ± SE from six mice. **P < 0.01 and ***P < 0.001 compared with the respective values in the same-day group. (Tukey’s test). (For interpretation of the references to colour in this figure legend, the reader is referred to the web version of this article.)

**Fig. 5:**
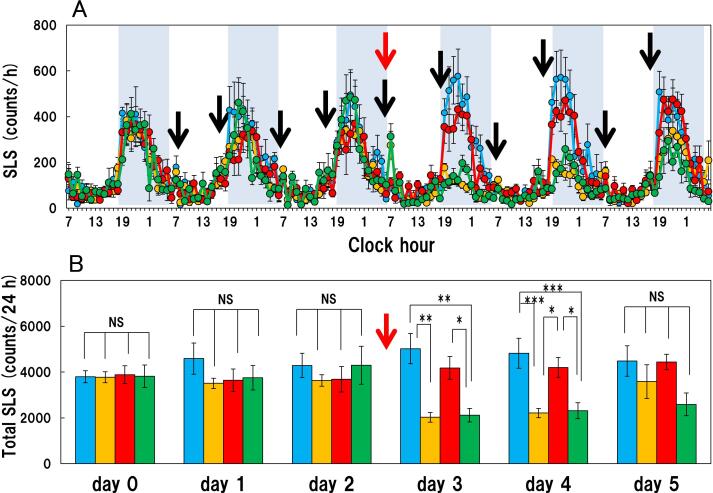
Effect of topically applied 1.0% capsaicin on IL-31-induced locomotor activity in BALB/c mice. (A) Short-lasting scratching induced (locomotor activity) by IL-31 was measured for 96 h in BALB/c mice. The black arrow indicates the site of injection of the vehicle or IL-31 (50 μg/kg, subcutaneously) every 12 h (7:00 am and 7:00 pm) for 96 h. The lateral axis indicates the clock hour, and the shaded area represents the dark phase (7:00 pm to 7:00 am). The red arrow indicates the site of topical application of the vehicle or capsaicin. The blue line indicates saline + vehicle-treated group, the yellow line indicates saline + capsaicin treated group; the red line indicates IL-31 + vehicle-treated group; the green line indicates the IL-31 + capsaicin-treated group. (B) Total short-lasting scratching counts measured 24 h before administering IL-31 (day 0) and on day 1 (0–24 h), day 2 (25–48 h), day 3 (48–72 h), day 4 (72–96 h) and day 5 (96–120 h) after administering IL-31. The blue column indicates saline + vehicle-treated group, the yellow column indicates saline + capsaicin treated group; the red column indicates IL-31 + vehicle-treated group; the green column indicates IL-31 + capsaicin-treated group. Each value represents the mean ± SE from six mice. **P < 0.01 and ***P < 0.001 compared with the respective values in the same-day group. (Tukey’s test). (For interpretation of the references to colour in this figure legend, the reader is referred to the web version of this article.)

There were no significant changes in the long-lasting scratching and short-lasting scratching counts during the experimental period in the vehicle (saline) treated group from day 0 (-24–0 h) - day 5 (120–144 h) after the vehicle administration (Fig. 4A, blue line; Fig. 4B, blue column). Repeated administration of IL-31 (50 μg/kg, subcutaneous), every 12 h (7:00 and 19:00) for 5 days gradually increased long-lasting scratching counts in BALB/c mice (Fig. 4A, red line; Fig. 4B, red column). The IL-31-induced increase in long-lasting scratching counts in the vehicle-applied group plateaued about 72 h after initiating the repeated IL-31 administration (Fig. 4B). The total long-lasting scratching counts on days 0 (pretreatment), 1, 2, 3, 4 and 5 after administration of IL-31 were 173.2 ± 40.6, 188.7 ± 12.1, 341.1 ± 41.1, 406.2 ± 39.8, 399.5 ± 52.7 and 380.7 ± 50.3 counts/24 h, respectively (Fig. 4B, red column). Total long-lasting scratching count was significantly increased from days 2–5 in the IL-31 treated-group compared to the counts in the saline treated group. Therefor we applied capsaicin (1.0%) alone at 72 h administrating IL-31(Fig. 4A, B; red arrows). Capsaicin significantly suppressed saline-treated or IL-31-induced long-lasting scratching from days 3–5 (Fig. 4A, B; yellow and green column). Repeated administration of IL-31 did not increase the short-lasting scratching counts significantly. The total short-lasting scratching counts on days 0, 1, 2, 3, 4 and 5 measured at 24 h-intervals after administration of IL-31 were 4244 ± 253, 3642 ± 492, 3685 ± 554, 3494 ± 275, 4203 ± 435 and 4444 ± 338 counts/24 h, respectively (Fig. 5A, red line). However, capsaicin significantly suppressed short-lasting scratching from day 3–4 in the saline or IL-31 treated groups (Fig. 5A, B; yellow and green columns).

### 3.5 Time course of the changes in the expression of cutaneous IL-31RA and of IL-31RA and TRPV1 mRNA in the DRG of BALB/c following the topical application of capsaicin

**Fig. 6:**
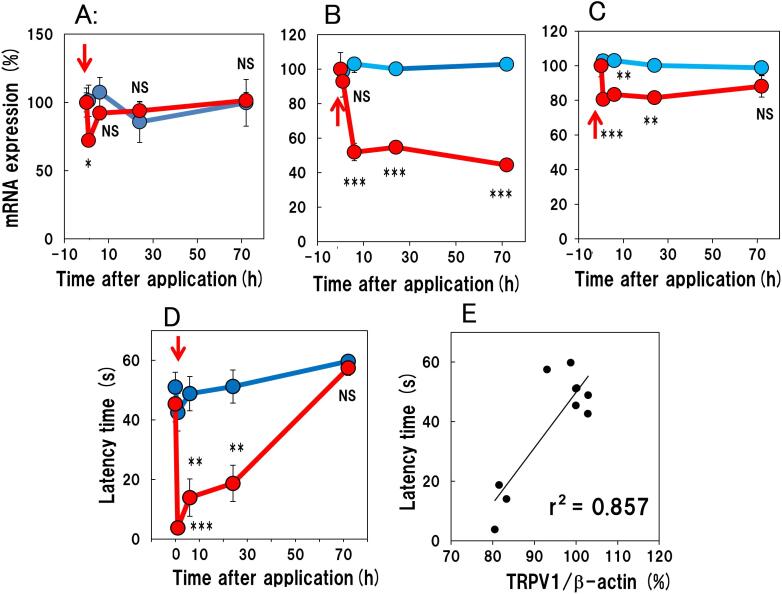
Time course of the changes in the expression of cutaneous IL-31RA mRNA and IL-31RA and TRPV1 mRNA expression in the DRG of 1.0% capsaicin treated BALB/c mice. Expression of cutaneous (A) IL-31RA mRNA (cIL-31RA). (B) IL-31RA mRNA in the DRG (dIL-31RA) and (C) TRPV1 mRNA in the DRG (dTRPV1). (D) Pain threshold after capsaicin application measured using hot-plate test. (E) Correlation between the latency and expression of TRPV1 mRNA in the DRG. The blue line indicates the vehicle (ethanol)-treated group, while the red line indicates 1.0% capsaicin-treated group. Each value represents the mean ± SE from six mice. N.S., not significant. * P < 0.05, **P < 0.01 and ***P < 0.001 compared with the corresponding values of vehicle-treated group (Student’s *t* test). (For interpretation of the references to colour in this figure legend, the reader is referred to the web version of this article.)

There were no significant changes in the cutaneous IL-31RA and DRG TRPV1 mRNA expression over the experimental period in the vehicle treated group (Fig. 6A - C; blue lines). Cutaneous IL-31 mRNA expression was decreased 24 to 72 h after applying 1.0% capsaicin but not significantly (data not shown). Cutaneous IL-31RA mRNA expression was decreased temporarily 1 h after applying capsaicin but soon recovered the basal level, which was not different from its expression in the vehicle-treated group (Fig. 6A, red line). IL-31RA mRNA expression in the DRG was significantly decreased from 6 to 72 h after applying capsaicin compared with the corresponding value of the vehicle-treated group (Fig. 6B, red line). TRPV1 mRNA expression in the DRG was significantly decreased from 1 to 24 h after applying capsaicin, and the decrease was not significant at 72 h after application compared with the corresponding expression in the vehicle-treated group (Fig. 6C).

### 3.6 Time course of the changes in the pain threshold of BALB/c mice following the topical application of capsaicin

The paw withdrawal latency measured using hot-plate test was significantly shortened immediately after applying 1.0% capsaicin and then gradually returned to the baseline 72 h later (Fig. 6D). The decreased IL-31RA mRNA expression in the DRG was correlated with decreased pain threshold. There was a significant correlation between the latency and the expression of TRPV1 mRNA (Fig. 6C) in the DRG (Fig. 6E, r2 = 0.782).

## 4 Discussion

There are two kinds of itching in mice, pruritogens (i.e., histamine intradermal injection) caused itching and IL-31 (whole body administration) caused itching. Moreover, the difference in these itches emerges for action of capsaicin. Topically applied capsaicin-induced desensitization leads to a loss of sensitivity to pain induced by noxious heat or capsaicin and a loss of histamine-induced itching (Tóth-Kása et al., 1986; Fuchs et al., 2000). In contrast, although cowhage-induced itching is completely suppressed in capsaicin-desensitized skin, the itching sensation caused by intradermal histamine is only slightly decreased, suggesting that there are different mechanisms between cowhage- and histamine-induced itching (Johanek et al., 2007). In this study, capsaicin completely suppressed spontaneous scratching in skin-lesioned NC/Nga mice and IL-31-induced scratching in BALB/c mice. We have previously reported that IL-31 is one of the most important factors that induces itching and promotes scratching in NC/Nga mice (Arai et al., 2013, Arai et al., 2015). Our previous data also showed that itching patterns were different in scratching behavior induced by IL-31- and other pruritogens (i.e., histamine, serotonin, compound 48/80, substance P, PAR2). A single intradermal injection of IL-31 elicited long-lasting scratching but short-lasting scratching that began gradually about 2 h after injection and persisted over 24 h. In contrast, histamine (Takano et al., 2004), serotonin (Akiyama et al., 2009a), compound 48/80 (Inagaki et al., 2002) substance P (Scholzen et al., 2004) or PAR2 (Akiyama et al., 2009a) elicited short-lasting scratching but not long-lasting scratching, which began immediately after the intradermal injection and persisted for at least 30 min. Moreover, after the intradermal injection of histamine, skin-lesions were not observed in mice. Conversely, long-lasting scratching was increased in skin-lesioned NC/Nga mice, and there was a marked difference, especially in those with duration over 1.0 s. Therefore, we selected the long-lasting scratching count as an indicator of the true itch. Although, we used long-lasting scratching as an indicator of the itch-associated scratching behavior, long-lasting scratching is not observed during histamine-induced itching.

It is well known that pain inhibits itching (Davidson and Giesler, 2010). It is possible that the antipruritic effect of capsaicin is induced by pain stimuli activating TRPV1. In the current study, the inhibitory action of capsaicin on long-lasting scratching was not only strong but also very persistent over long periods. Moreover, the long-lasting scratching counts were closely correlated with IL-31RA mRNA in the DRG. These results indicate that the activation of TRPV1 and inhibition of IL-31RA mRNA expression in the DRG may mediate the antipruritic effects of capsaicin. The decrease in TRPV1 mRNA expression in the DRG triggered by capsaicin is hypothesized to be a response to the onset of TRPV1-stimulatd pain. The application of 0.1 or 1.0% capsaicin suppressed both long-lasting scratching and short-lasting scratching for 24 h after treatment-, with 1.0% capsaicin suppressing itching from 0 to 72 h. The expression of IL-31RA significantly decreased after 72 h following capsaicin application, but the expression of cutaneous IL-31RA and TRPV1 were not changed compared with the corresponding expression levels in the vehicle-treated group. These data suggest that the suppression of long-lasting scratching and short-lasting scratching may be dependent on the inhibition of IL-31RA mRNA expression in DRG and not on the inhibition of TRPV1 mRNA expression.

Previously, we found that the intensity of IL-31-induced long-lasting scratching in skin-lesioned NC/Nga mice is influenced by the expression of IL-31RA in the DRG but not of cutaneous IL-31RA (Arai et al., 2015). Repeated administration of IL-31 (50 μg/kg, subcutaneous) gradually increased long-lasting scratching and DRG IL-31RA mRNA expression, and these reached a plateau after the administering IL-31 for 72 h in NC/Nga and BALB/c mice. Close correlations between long-lasting scratching and DRG IL-31RA mRNA expression were observed. IL-31-induced scratching behavior in BALB/c mice, with only long-lasting scratching counts increased. In skin-lesioned NC/Nga mice, several inflammatory mediators (i.e., histamine, serotonin, bradykinin, substance P and so on) not only IL-31, act on skin to increasing not only long-lasting scratching, but also short-lasting scratching. However, capsaicin strongly suppressed long-lasting scratching and short-lasting scratching immediately after application and over 72 h later in BALB/c mice. It is a well-known reaction that a pain suppresses the locomotor activities (short-lasting scratching). This result suggests that the suppressing effect of short-lasting scratching was triggered by capsaicin induced onset of TRPV1-stimulatd pain, since the decreased DRG TRPV1mRNA expression duration. These results show that suppression of scratching may occur due to the inhibited expression of IL-31RA mRNA in the DRG. In contrast, the expression of TRPV1 mRNA in the DRG was restored and did not correlate with the suppression of long-lasting scratching after the application of capsaicin.

Capsaicin has been widely used as a reliable research tool for inducing pain, which is mediated by the activation of TRPV1 in nociceptive afferent terminals (Caterina et al., 1997). It is probable that the pain that is partially induced through the activation of TRPV1 by capsaicin is, in turn, responsible for suppressing itching sensations. Paradoxically, the application of capsaicin not only produces transient burning pain but also induces analgesia to neuropathic pain. The mechanism underlying the opposing actions of capsaicin with respect to the onset of pain and induction of analgesia has not yet been explained. The application of capsaicin causes desensitization of TRPV1 (Joseph et al., 2013), inhibition of nociceptor firing (Ma et al., 2015), and decrease in mechanotransduction (Borbiro et al., 2015). These early effects of capsaicin on the function of primary afferents might potentially contribute to the analgesic effects immediately after capsaicin application. However, these effects may be unrelated to the suppression of itching considering that there are no reports that analgesic action inhibits itching more than 72 h following capsaicin application. It is reported that IL-31RA develops on C-fibers (Bando et al., 2006), and there may be TRPV1 and some kind of interaction to expression on the same C-fiber. We had previously reported that IL-31-induced itching partially participates in the analgesic effect of morphine in the periphery during the pain restraint mechanism (Tsuji et al., 2019). Overall, our study findings suggests that the decrease in DRG IL-31RA after the application of capsaicin participates in the onset of pain induction by capsaicin in the late phase. However, it is difficult to use an IL-31RA expression inhibitor, such as tacrolimus, along with TRPV1 stimulation as a therapeutic drug, as a patient with AD experienced severe pain immediately after its application (Pereira et al. (2010)). If an IL-31RA expression inhibitor can be used in the DRG without the TRPV1 stimulation, it will likely become a suitable therapeutic drug for AD.

In conclusion, the strong and prolonged antipruritic action for IL-31-induced itching of capsaicin was caused by desensitization of C-fibers, and, in addition, the long-lasting inhibition of IL-31RA mRNA expression in the DRG.

CRediT authorship contribution statement

**I. Arai:** designed the study, conducted the study, collected the data and prepared the manuscript, **M. Tsuji:** collected the data and helped in the preparation of the manuscript, **H. Takeda:** conducted the study and helped in the preparation of the manuscript, **N. Akiyama:** contributed essential reagents, **S. Saito:** conducted the study and helped in the preparation of the manuscript. All authors read and approved the final manuscript.

## Declaration of Competing Interest

This research did not receive any specific grant from funding agencies in the public, commercial, or not-for-profit sections.
